# Targeted Metabolic and In-Silico Analyses Highlight Distinct Glucosinolates and Phenolics Signatures in Korean Rapeseed Cultivars

**DOI:** 10.3390/plants10102027

**Published:** 2021-09-27

**Authors:** Joonyup Kim, Soo In Sohn, Ramaraj Sathasivam, Allah Jurio Khaskheli, Min Cheol Kim, Nam Su Kim, Sang Un Park

**Affiliations:** 1 Department of Horticultural Science, Chungnam National University, 99 Daehak-ro, Yuseong-gu, Daejeon 34134, Korea; aajkhaskheli@gmail.com; 2 Biosafety Division, Department of Agricultural Biotechnology, Jeonju 54874, Korea; sisohn@korea.kr; 3 Department of Crop Science, Chungnam National University, 99 Daehak-ro, Yuseong-gu, Daejeon 34134, Korea; ramarajbiotech@gmail.com (R.S.); mincheol2641@naver.com (M.C.K.); 4 Korea Research Institute of Bioscience and Biotechnology, 30 Yeongudanji-ro, Ochang-eup, Cheongju 28116, Korea; kns917555@kribb.re.kr; 5 Department of Smart Agriculture Systems, Chungnam National University, 99 Daehak-ro, Yuseong-gu, Daejeon 34134, Korea

**Keywords:** *Brassica napus*, rapeseed, glucosinolates, phenolics, biosynthesis pathway, *cis*-regulatory elements, metabolic profiles

## Abstract

Rapeseed is an economically important oilseed crop throughout the world. We examined the content and composition of glucosinolates (GSLs) and phenolics in the sprouts of seven Korean cultivars. A total of eight GSLs that include four aliphatic GSLs (AGSLs) (progoitrin, gluconapin, gluconapoleiferin, and glucobrassicanapin) and four indole GSLs (IGSLs) (4-methoxyglucobrassicin, 4-hydroxyglucobrassicin, neoglucobrassicin, and glucobrassicin) were identified in these cultivars. Of the total GSLs, the highest level was detected for progoitrin, while the lowest level was identified for glucobrassicanapin in all the cultivars. Phenolics that include chlorogenic acid, catechin hydrate, 4-hydroxybenzoic acid, gallic acid, ferulic acid, *p*-coumaric acid, epicatechin, caffeic acid, rutin, quercetin, *trans*-cinnamic acid, benzoic acid, and kaempferol were present in all the cultivars. Of these, rutin was identified with the highest level while *trans*-cinnamic acid was identified with the lowest level in all the cultivars. Cluster analysis revealed the unique metabolic signature of eight GSLs and thirteen phenolics for the seven cultivars of rapeseed, which implies that genomic commonality and variability resulted from the previous breeding program. Further, gene expression and *cis*-regulatory elements suggest that the biosynthesis of GSLs and phenolics of these cultivars appears to be regulated through transcription factors associated with stress responses, phytohormones, and cellular growth.

## 1. Introduction

Rapeseed (*Brassica napus* L.) is one of the most important edible oil crops in the world, with high nutritional value for humans and a protein source for livestock [1,2]. It is also a source of alternative fuel and edible oil in Korea with a low self-sufficiency rate that has a high development value. Among vegetables, the Brassicaceae family is well-known for its excellent dietary value and has received a lot of attention in recent years. The Brassicaceae family includes a diverse group of vegetables widely consumed in the world. Previous studies reported that the seeds of *Brassicaceae* vegetables are rich in unsaturated fatty acids, which have diverse health-promoting effects [3].

Plant secondary metabolites are small-molecule products that play roles in plant survival and reproduction resulting from adaptation to abiotic and biotic stress [4]. Plant secondary metabolites are derived from the structural units synthesized in primary and intermediate metabolism, such as aromatic amino acids from the shikimate pathway and isopentenyl diphosphate from the isoprenoid pathway diphosphate [5,6]. They can be divided into four major classes: terpenoids, and sulfur-containing compounds such as glucosinolates (GSLs), alkaloids, and phenolics [7,8]. Over recent decades, plant secondary metabolites such as GSLs have been gaining great interest because of their cancer-preventive properties for humans as well as their nutritional value [2,9,10,11], which evoked the demand for the breeding of functional cultivars and industrial applications [12].

In the past years, GSLs have been attracting interest due to the biocidal and anticancer activity of their hydrolyzed products, such as epithionitriles, isothiocyanates (ITCs), nitriles, oxazolidines, and thiocyanates [13,14,15]. GSLs are categorized into three classes (aliphatic, indolic, and aromatic GSLs), and chemically, GSLs are hydrolyzed by myrosinase to produce glucose and unstable aglycone that further undergoes metabolic reconfiguration to give rise to various biologically active compounds in response to environmental stress [16]. Plant GSLs are sulfur- and nitrogen-containing specialized metabolites exclusively identifiable in the order of *Brassicales* [17]. Human intake of these vegetables rich in the ITCs is known to help reduce the risk of cancer [18,19]. In addition, it is well-known that GSLs play a crucial role in the defense and survival system of the *Brassicaceae* [11,20], and it has been reported that GSLs might act as a sink for nutrients, such as nitrogen and sulfur [21,22,23]. Nonetheless, the physiological significance and mechanistic regulations of GSLs and their degradation products in many plants are not fully understood. Previous studies have demonstrated that there are many factors, including genotypes, growing conditions, tissue types, and harvest time, that affect the contents and chemical compositions of GSLs in diverse *Brassicaceae* vegetables [7,16].

Phenolic compounds are also essential for the survival of plants in adverse environments [20,24,25]. Phenolic compounds are widely present in many plant species and have an important role in plant defense responses [25,26]. In addition, the beneficial effects of phenolic compounds identified in the *Brassicaceae* are linked to the antioxidant activity that is used for human health and disease prevention [11,24,27]. Equivalent to those GSLs, the content and the pharmacological functions of the phenolic compounds depend on pre- and post-harvest factors, such as environmental and agronomic conditions, variety selection, maturity stage, and extraction procedures [28]. Further, depending on the tissue type studied, even from the same *Brassicaceae* plant (e.g., kale), the content and composition of phenolics can differ [29].

Plant sprouts are an important source of essential as well as non-essential organic molecules (carbohydrates, proteins, minerals, and vitamins) associated with health beneficial properties (antioxidant activity, anti-cancer properties, and antibiotic effects) [30]. It has been shown that plant sprouts can help alleviate human diseases [31,32] as they contain many healthcare compounds, including GSLs, polyphenols, sterols, and vitamins [30].

To understand the metabolic features of rapeseed cultivars associated with seed germination, we examined the content and composition of GSLs and phenolics in the sprouts of seven Korean winter cultivars using targeted metabolomics. In addition, we analyzed the *cis*-regulatory elements for the genes associated with biosynthetic pathways of GSLs and phenolics implemented with gene expression analysis. Resultant data from our study uncover those unique metabolic signatures embedded in these cultivars and will be instrumental for future studies and for breeding functional cultivars of Korean rapeseed.

## 2. Results

### 2.1. Examination of Glucosinolates (GSLs) in the Seven Korean Winter Cultivars of Rapeseed

**Figure 1:**
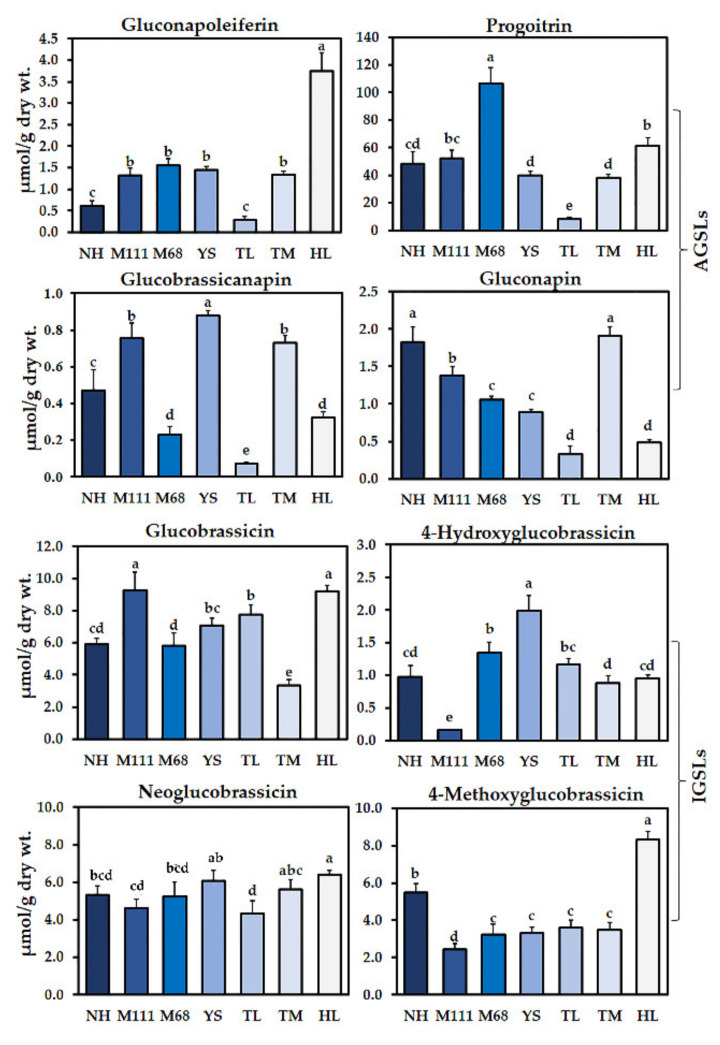
Determination of GSLs in the sprouts of seven Korean rapeseed cultivars. Aliphatic GSLs (AGSLs) and indolic GSLs (IGSLs) identified in the cultivars of Naehan (NH), Mokpo111 (M111), Mokpo68 (M68), Youngsan (YS), Tamla (TL), Tammi (TM), and Hanla (HL) are represented. Different letters indicate significant differences detected for the GSLs between the cultivars analyzed by ANOVA-DMRT (*p* < 0.05). The results shown represent the mean values of three biological replicates with standard deviation.

The content and composition of GSLs were analyzed in the seven cultivars of rapeseed (Naehan, Mokpo111, Mokpo68, Youngsan, Tamla, Tammi, and Hanla). A total of eight GSLs that include four aliphatic GSLs (AGSLs) (progoitrin, gluconapin, gluconapoleiferin, and glucobrassicanapin) and four indole GSLs (IGSLs) (4-methoxyglucobrassicin, 4-hydroxyglucobrassicin, neoglucobrassicin, and glucobrassicin) were identified in the seven rapeseed cultivars (Figure 1). In all the cultivars, the amount of total GSLs showed a significant difference (*p* ≤ 0.05), and it ranges from 23.76 (Tamla) to 124.99 (Mokpo68) μmol/g of dry weight (wt.) (Supplementary Figure S1 and Table S1). Of the total GSLs examined for all seven cultivars, the highest level of GSL was detected for progoitirin, while the lowest level of GSL was identified for glucobrassicanapin, except for the Mokpo111 cultivar (Figure 1, Supplementary Figure S1 and Table S1). Although the amount of GSLs was varied, the level of IGSLs (4-methoxyglucobrassicin, 4-hydroxyglucobrassicin, neoglucobrassicin, and glucobrassicin) was found to be retained at a lower level compared with progoitrin, but sustained at a relatively higher level than the rest of the AGSLs in all the cultivars (Figure 1 and Supplementary Table S1).

### 2.2. Determination of Phenolic Compounds in the Seven Korean Winter Cultivars of Rapeseed

**Figure 2:**
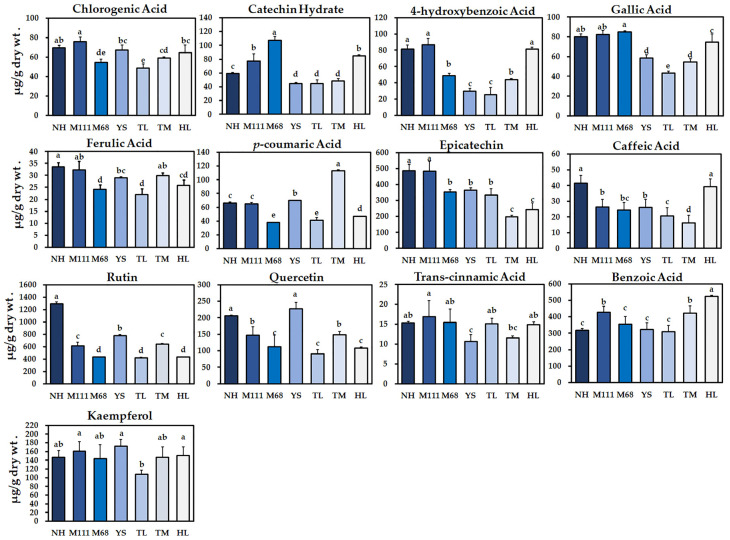
Determination of phenolic compounds in the sprouts of seven Korean rapeseed cultivars. Different letters indicate significant differences detected for the phenolic compounds between the cultivars analyzed by ANOVA-DMRT (*p* < 0.05). The results shown represent the mean values of three biological replicates with standard deviation.

Phenolic compounds that may be present in the sprouts of seven cultivars of rapeseed were analyzed using HPLC. A total of 13 phenolic compounds, including chlorogenic acid, catechin hydrate, 4-hydroxybenzoic acid, gallic acid, ferulic acid, *p*-coumaric acid, epicatechin, caffeic acid, rutin, quercetin, *trans*-cinnamic acid, benzoic acid, and kaempferol, were detected and quantified in all seven cultivars (Figure 2). The total phenolic compounds in all the examined cultivars of rapeseed were detected in a range from 1521.42 (Tamla) to 2893.84 (Naehan) µg/g of dry wt. (Supplementary Figure S1 and Table S2). Interestingly, among all the individual phenolic compounds, rutin showed the highest level in all the cultivars and it was significantly highest in the Naehan cultivar (1292.46 µg/g dry wt.) (Figure 2). The second most predominant phenolic compounds identified were benzoic acid, followed by epicatechin in the seven cultivars (Figure 2 and Supplementary Table S3). Of the total phenolic compounds, the lowest level of a phenolic compound identified was *trans*-cinnamic acid in all the cultivars, particularly in the Youngsan cultivar (10.69 µg/g dry wt.) (Supplementary Figure S1 and Table S3).

### 2.3. Relationships of GSLs and Phenolics in the Seven Korean Winter Rapeseed Cultivars

In total, 21 metabolites were identified in the 7 cultivars of rapeseed. Among these, the amounts of GSLs, progoitrin, glucobrassicin, 4-methoxyglucobrassicin, and neoglucobrassicin were significantly higher, while phenolic compounds such as rutin, epicatechin, and benzoic acid were significantly higher in all the seven rapeseed cultivars (Figure 1 and Figure 2). From these results, it was found that all the seven rapeseed cultivars consist of similar higher levels of these individual GSLs and phenolic compounds, although, the content of each GSL and phenolic content varied based on the cultivars.

**Figure 3:**
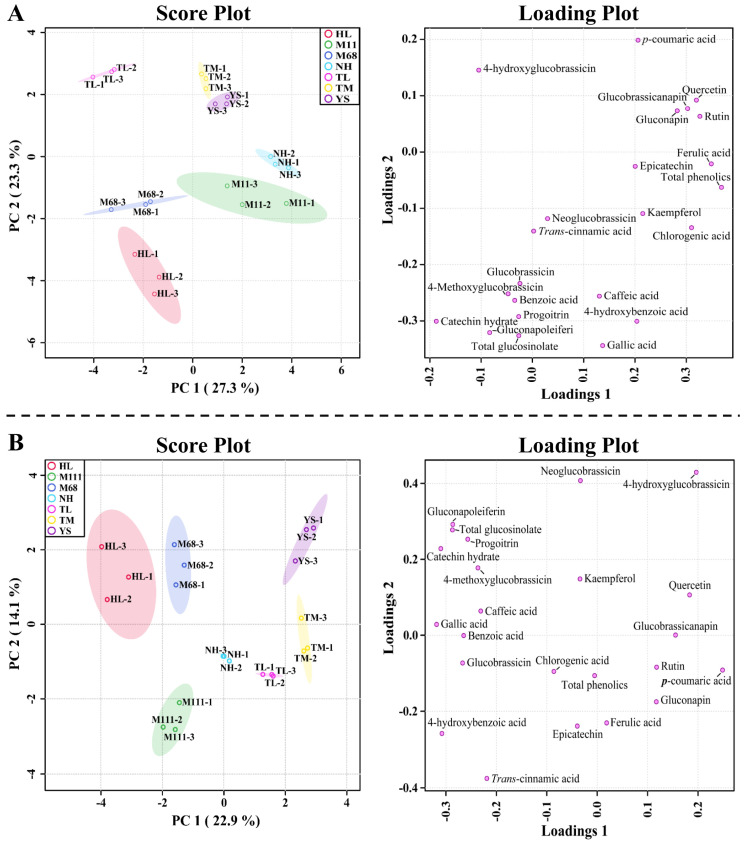
Score plots and loading plots of the (**A**) principal component analysis (PCA) and (**B**) partial least squares-discriminant analysis (PLS-DA) results obtained from 21 secondary metabolites from the sprouts of seven Korean rapeseed cultivars.

The above result was further supported by the PCA analysis. The PCA plot showed that these metabolites differed between all the seven rapeseed cultivars based on the two main components analysis (27.3% and 23.3%, respectively) (Figure 3). In addition, all the seven rapeseed cultivars were clearly separated, and the most important metabolites for clear separation of PC1 were ferulic acid, rutin, quercetin, chlorogenic acid, glucobrassicanapin, and gluconapin, and the related eigenvector values were 0.34893, 0.32665, 0.31981, 0.31037, 0.30266, and 0.28239, respectively. The eigenvector values of catechin hydrate, 4-hydroxyglucobrassicin, gluconapoleiferin, 4-methoxyglucobrassicin, and benzoic acid were −0.18756, −0.10467, −0.083437, −0.047537, and −0.034542, respectively (Figure 3). Moreover, PLS-DA also supports the PCA result in which the PLS-DA result displayed a clear separation between the cultivars based on two components’ variances (22.9% and 14.1%, respectively). Collectively, the clear separation demonstrated from the PCA and PLS-DA analyses is likely due to changes in the GSL and phenolic content specific to these cultivars.

### 2.4. Metabolic Profiling of Identified Metabolites from Seven Korean Cultivars

**Figure 4:**
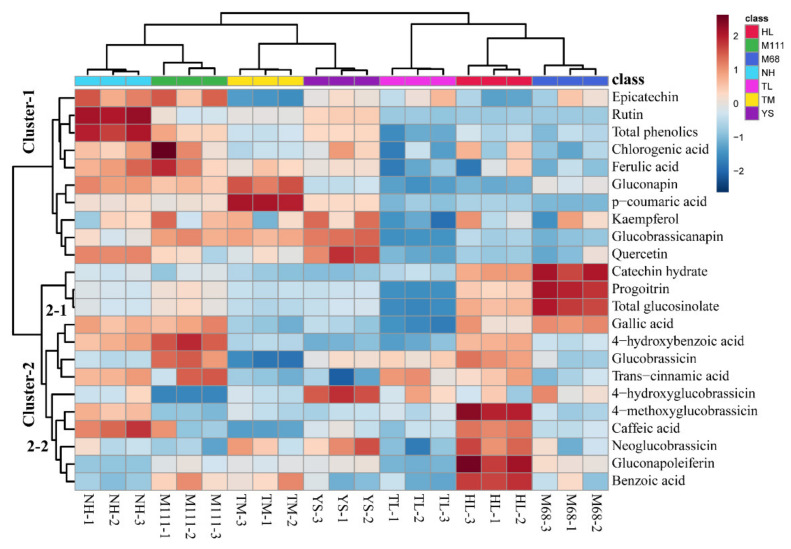
Visualization of hierarchical clustering and heatmap for 8 GSLs and 13 phenolic compounds identified in the sprouts of 7 Korean rapeseed cultivars. Relative abundance levels within a metabolite are shown in red, white, and blue colors for above average, average, and below average values, respectively. Clustering analysis and heatmap based on *t*-tests and auto-scaling normalization for the metabolites were performed using MetaboAnalyst 5.0 (https://www.metaboanalyst.ca, accessed on 20 August 2021).

To further understand the relationship of metabolites identified in the 7 cultivars, the metabolite profiles of 8 GSLs and 13 phenolic compounds in the sprouts of 7 rapeseed cultivars were visualized using hierarchical clustering and heatmap (Figure 4). Relative abundance levels within a metabolite are shown in red, white, and blue colors for above average, average, and below average values, respectively. As shown in Figure 4, regardless of classes of metabolites (i.e., GSLs and phenolics), each cultivar contains the unique feature of metabolites embedded in the cultivars examined. The heatmap is divided into 2 major clusters, namely cluster 1 and cluster 2, whereas cluster 2 is divided into 2 subclusters (2-1 and 2-2) (Figure 4). The GSLs and phenolic compounds were clustered into three groups that delineate above average (group 2-1), average (group 2-2), and below average (group 1). Specifically, the subcluster 2-1 was found to be abundant in Mokpo68 and lacking in Tamla compared to other cultivars. In the subcluster 2-2, 4-methoxyglucobrassicin, caffeic acid, neoglucobrassicin, gluconapoleiferin, and benzoic acid were most abundant in Hanla, and least abundant in Tamla. As for the cluster 1, epicatechin, rutin, and total phenolics were most abundant in Naehan, and found to be least abundant in Tamla and Hanla. In addition, most of the metabolites identified in the seven cultivars for AGSLs and IGSLs were separated into distinct groups (e.g., AGSLs in all groups of clusters and IGSLs in groups 2-1 and 2-2). Regarding the phenolic compounds, metabolites for seven cultivars were separated into four groups (Figure 4). Group 1 consists of epicatechin, rutin, chlorogenic acid, ferulic acid, p-coumaric acid, kaempferol, and quercetin, whereas group 2-1 consists of catechin hydrate, gallic acid, 4-hydroxybenzoic acid, and trans-cinnamic acid. In group 2-2, caffeic acid and benzoic acid were clustered. This result demonstrates the unique metabolite signature inherent to the Korean rapeseed cultivars.

### 2.5. Gene Expression Analysis of Biosynthetic Pathways for GSLs and Phenolics

**Figure 5:**
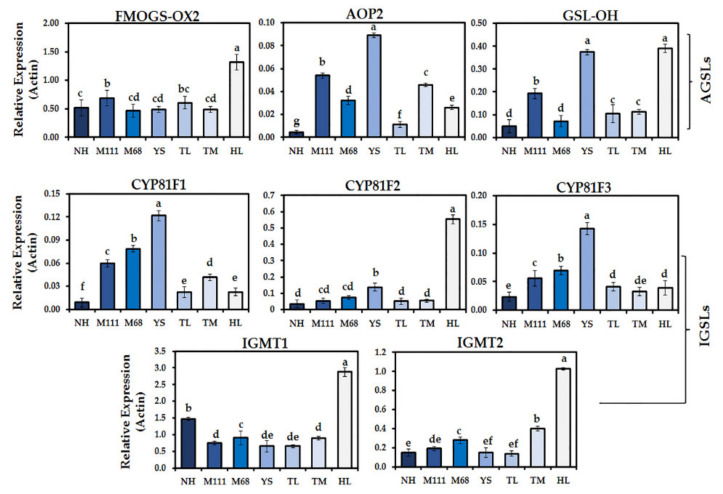
Transcript levels of selected genes in the biosynthesis for the GSLs in the sprouts of seven Korean rapeseed cultivars. Transcript levels of each gene associated with the GSLs pathway were compared to that of rapeseed *ACTIN2* (*BnaACT2*). Data represent the mean ± standard deviation for three biological replicates with at least two technical replicates. Different letters indicate significant differences detected for the GSLs between the cultivars analyzed by ANOVA-DMRT (*p* < 0.05).

**Figure 6:**
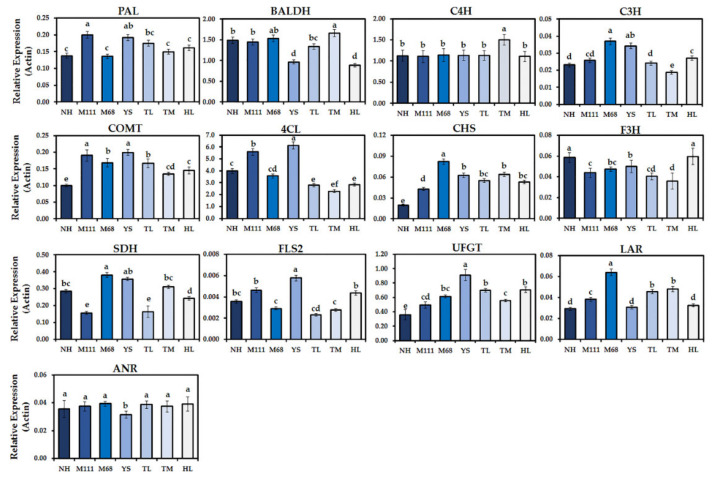
Transcript levels of selected genes in the biosynthesis for the phenolic compounds in the sprouts of seven Korean rapeseed cultivars. Transcript levels of each gene associated with the phenolics pathway were compared to that of rapeseed *ACTIN2* (*BnaACT2*). Data represent the mean ± standard deviation for three biological replicates with at least two technical replicates. Different letters indicate significant differences detected for the phenolic compounds between the cultivars analyzed by ANOVA-DMRT (*p* < 0.05).

To understand the transcriptional changes associated with the biosynthesis of GSLs and phenolics compounds in the seven Korean winter cultivars, we examined the expression of selected genes immediately linked to the changed metabolites (Figure 5 and Figure 6). We measured the expression levels of eight different GSL biosynthetic genes in the seven cultivars. Of the genes analyzed for the biosynthesis of AGSLs, flavin-containing monooxygenase 2 (*FMO_GS-OX2_*) had the highest level of expression, followed by glucosinolate hydroxylase (*GSL-OH*) and 2-oxoglutarate-dependent dioxygenase (*AOP2*) (Figure 5). As for the biosynthetic genes of IGSLs, indole GSL *O*-methyltransferase 1 (*IGMT1*) displayed the highest level of expression, followed by three cytochrome P450 81F genes (i.e., *CYP81F1*, *CYP81F2*, and *CYP81F3*) (Figure 5). Regarding the biosynthetic genes associated with phenolic compounds, we examined the expression of phenylalanine ammonia-lyase (*PAL*), sorbitol dehydrogenase (*SDH*), benzaldehyde dehydrogenase (*BALDH*), cinnamic acid 4-hydroxylase (*C4H*), *p*-coumarate 3-hydroxylase (*C3H*), caffeic acid 3-*O*-methyltransferase (*COMT*), 4-coumarate CoA ligase (*4CL*), chalcone synthase (*CHS*), flavanone 3-hydroxylase (*F3H*), flavonol synthase2 (*FLS2*), UDP-glucose:flavonoid 3-O-glucosyltransferase (*UFGT*), anthocyanidin reductase (*ANR*), and leucoanthocyanidin reductase (*LAR*) in the sprouts of seven rapeseed cultivars (Figure 5). Overall, patterns of gene expression were relatively correlated with metabolite changes identified in this study (Figure 2, Figure 4, and Figure 6). In particular, the level of expression for *UFGT* and *BALDH* was positively correlated with the corresponding changed metabolites (Figure 2, Figure 4, and Figure 6). Interestingly, gene expression of *4CL* was highly upregulated in the seven cultivars. Among others, the expression of *FLS2* was greatly downregulated in all the cultivars (Figure 6).

### 2.6. In Silico Analysis Highlights the cis-Regulatory Elements and Transcriptional Regulation Associated with Metabolite Changes

**Figure 7:**
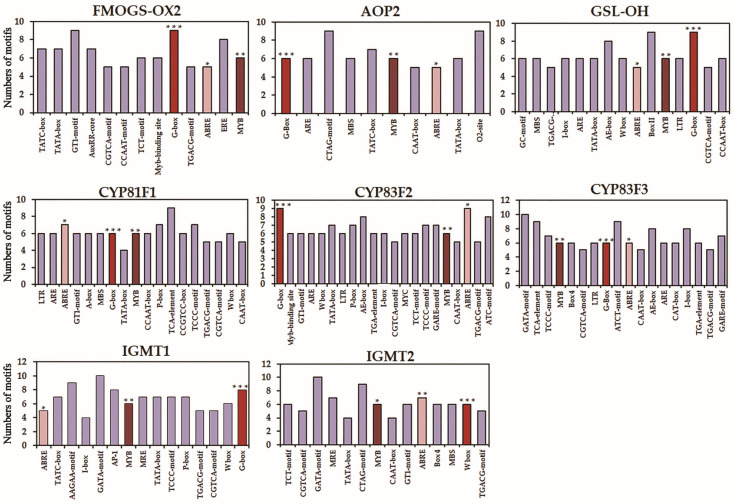
*Cis*-regulatory elements and transcription factor (TF) binding motifs identified in the biosynthetic genes of GSLs. In addition to the elements involved in the general transcriptional regulation of genes (TATA- and CAAT-box), *cis*-regulatory elements or binding motifs linked to the biosynthesis of GSLs included phytohormones (auxin, gibberellin, jasmonic acid, and abscisic acid), stress (anaerobic induction, light response, and low-temperature response), and TFs (MYC and MYB). Asterisks denote the common motifs associated with transcription factors G-box (***, bZIPs and bHLHs), MYB (**), and ABRE (*, abscisic acid response).

**Figure 8:**
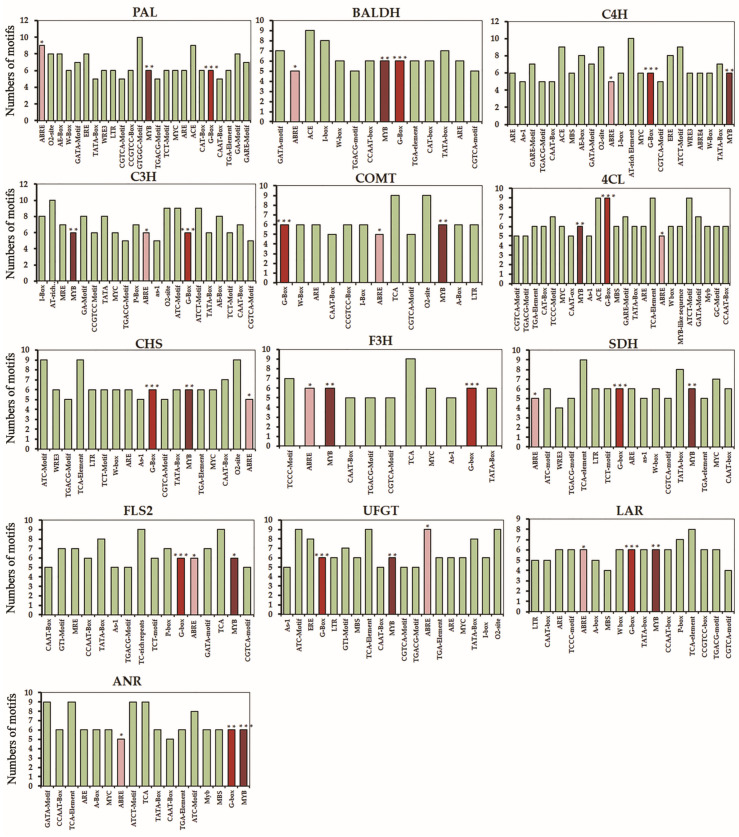
*Cis*-regulatory elements and transcription factor (TF) binding motifs identified in the biosynthetic genes of phenolic compounds. In addition to the elements involved in the general transcriptional regulation of genes (TATA- and CAAT-box), *cis*-regulatory elements or binding motifs linked to the biosynthesis of phenolic compounds included phytohormones (auxin, gibberellin, jasmonic acid, and abscisic acid), stress (anaerobic induction, light response, and drought response), and TFs (MYC and MYB). Asterisks denote the common motifs associated with transcription factors G-box (***, bZIPs and bHLHs), MYB (**), and ABRE (*, abscisic acid response).

The *cis*-regulatory elements of biosynthetic genes for GSLs and phenolics were examined (Figure 7 and Figure 8, Supplementary Table S3). As predicted, the length of *cis*-regulatory elements was 6–10 bp in the genes related to the pathway of GSLs, and 6–13 bp for the genes related to the phenolic compounds. The identified *cis*-regulatory elements are divided into different categories, in which the stress-responsive *cis*-elements were over-represented, that include the light-responsive element accounting for 34%. The number of different *cis*-regulatory elements involved in the biosynthetic pathway of GSLs and phenolic compounds was high. Selected genes related to the biosynthesis of GSLs and phenolic compounds revealed that in addition to the regulatory elements linked to the general transcriptional regulation of genes (i.e., TATA- and CAAT-box), the *cis*-regulatory elements or binding motifs for the biosynthesis of GSLs and phenolic compounds included phytohormones (auxin, gibberellin, jasmonic acid, and abscisic acid), stress (anaerobic induction, light response, low-temperature response, and drought response), and specific transcription factors (MYC and MYB). Of interest, the common motifs associated with G-box (bZIPs and bHLHs), MYB, and ABRE (abscisic acid response) were commonly predicted in the biosynthetic genes for both GSLs and phenolic compounds analyzed in this study (Figure 7 and Figure 8, Supplementary Table S3). Functional relationships of these regulatory elements in the regulation of GSLs and phenolic compounds for these Korean rapeseed cultivars remain to be studied.

## 3. Discussion

The *Brassicaceae* family represents excellent sources for various nutrients and phytochemicals. It is well-known that the *Brassicaceae* family contains diverse phytochemicals with beneficial biological properties that can contribute to human health [31] and plant survival [4], which have attracted extensive breeding programs and industrial interests over the past years [32]. *Brassicaceae* produce specialized metabolites such as GSLs, phenolics, tocopherols, and carotenoids [31]. In addition to these metabolites, the *Brassicaceae* plant including rapeseed is consumed as a major source of edible, industrial, and biofuel oil [33]. Moreover, it has been demonstrated that the content and composition of the above-mentioned metabolic features vary depending on numerous factors, from pre- to post-harvest conditions [7,16,28,34,35].

In the current study, we examined the content and composition of 16 GSLs, namely: aromatic compounds (gluconasturtiin), IGSLs (4-hydroxyglucobrassicin, 4-methoxyglucobrassicin, glucobrassicin, and neoglucobrassicin), AGSLs (glucoalyssin, glucoberteroin, glucobrassicanapin, glucoerucin, glucoiberin, gluconapin, gluconapoleiferin, glucoraphanin, glucoraphasatin, progoitrin, and sinigrin), and 13 phenolic compounds in the 7 winter cultivars of Korean rapeseed under normal germination conditions. We found that these seven Korean winter cultivars produce AGSLs and IGSLs but no aromatic GSLs (Figure 1). Although the level of metabolites differed, we identified four AGSLs (progoitrin, gluconapin, gluconapoleiferin, and glucobrassicanapin) and four IGSLs (4-methoxyglucobrassicin, 4-hydroxyglucobrassicin, neoglucobrassicin, and glucobrassicin) in the sprouts of rapeseed cultivars. Similar aliphatic and aromatic GSLs were also found in different tissue types (e.g., sprouts, seeds, and leaves of rapeseed) and even in response to biotic stress (i.e., arbuscular mycorrhizal) (Figure 1 and Supplementary Figure S2) [30,36,37,38]. Among the GSLs, the progoitrin was significantly higher in all the seven Korean rapeseed cultivars, and similar results were obtained in canola sprout of the rapeseed cv. Kizakino-natane [30,38]. Shahidi et al. [39] analyzed the individual GSLs content in six different canola seeds, and they also found progoitrin with the highest amount. In addition, they have reported that glucoberin was not detected in any of the canola seeds, which was consistent with our result. It is worthy to note that the minor amount of glucoerucin was detected by ammonia CI mass spectrometry in the canola seed, however, it was not detected in HPLC. Furthermore, Appelqvist [40] reported that this glucoerucin was present in *B. camperstris* but not in *B. napus* plants, which suggest that the accumulation of GSLs is organ- and species-specific.

Prior studies have shown that depending on the seed color and the seed developmental stages of rapeseed, the composition of phenolics such as flavonoids can vary [41]. In addition, growth conditions such as light quality can affect the content and composition of phenolics in the sprouts of rapeseed [30]. In this study, 13 phenolic compounds were detected, and similar phenolic compounds have been identified and reported in the previous studies of *Brassicaceae* seed coats [42,43,44,45]. Under normal germination conditions, we found that flavonoids such as rutin were detected as the most abundant phenolic compound, followed by benzoic acid catechin (epicatechin) (Figure 2 and Figure 4). Interestingly, although much variation exists for the total phenolic compounds as well as the total GSLs, the Tamla cultivar was found to have the lowest level for both total GSLs and phenolics (Supplementary Figure S1).

To further understand the relationship between the specialized metabolites and the seven cultivars, we employed cluster analysis (Figure 4). We found that each class of metabolites displayed a distinct grouping feature, which represents genomic commonality as well as minor variation that lies in the seven Korean rapeseed cultivars. Of note, most of the AGSLs and IGSLs identified in this study were grouped in the same cluster (Figure 4). In addition, metabolite profiles that were separated into each cultivar indicate that those seven cultivars appear to be bred in a similar breeding scheme. The result of our study further reveals that these rapeseed cultivars were developed to have a certain level of common metabolic features. Although they were clustered and grouped in similar metabolic categories with unique signatures, the level of each GSL and phenolic content varied, which opens the future breeding targets and schemes to develop more functional cultivars of Korean rapeseed. In addition, due to the different levels of GSLs and phenolics identified in the Korean rapeseed cultivars, we may have a better understanding on how we should approach or breed to engineer these rapeseed cultivars for a better beneficial effect for humans.

Most of the current knowledge on the genes involved in the biosynthesis of GSLs and their regulation can be attributed to studies using the model plant, *Arabidopsis thaliana* [46,47,48,49,50]. The biosynthesis of plant GSLs comprises three distinct steps: (1) side-chain elongation of amino acid, (2) metabolic reconfiguration of amino acid to the GSL core, and (3) side chain modification of the GSL core structure [51]. A prior study demonstrated that the conserved AtMYB115 and AtMYB118 that regulate the biosynthetic genes of GSLs are co-evolved and transcriptionally co-expressed with their target enzyme genes [52]. In addition, AtMYB115 and AtMYB118 may have regulatory roles in the three above-mentioned steps of biosynthesis of GSLs in *Arabidopsis thaliana*. To understand transcriptional changes that might have otherwise been associated with the detected GSLs in the seven Korean rapeseed cultivars, we examined the expression of the select genes immediately linked to the metabolite changes (i.e., step 3, side-chain modification of the GSL core structure) (Supplementary Figure S2) [46,51]. We found that the most highly upregulated gene related to AGSLs was *FMO_GS-OX2_*, followed by *GSL-OH* and *AOP2* (Figure 5 and Supplementary Figure S2). As for the IGSLs, the expression of IGMT1 was most highly upregulated, followed by *CYP81F1*, *CYP81F2*, and *CYP81F3* (Figure 5 and Supplementary Figure S2). On the contrary to the studies of *Arabidopsis thaliana*, the transcription factors that regulate the genes involved in the side-chain modification of the GSL core in these rapeseed cultivars are not determined at this moment. However, AtMYB transcription factors (AtMYB28, AtMYB29, and AtMYB76) are shown to play key roles in the regulation of AGSLs [47]. In addition, the transcription factors of the basic helix-loop-helix (bHLH) family, such as AtMYC2, AtMYC3, and AtMYC4, are found to be interacting with the above-mentioned AtMYBs to positively regulate the biosynthetic genes of AGSLs in *Arabidopsis thaliana* [47]. Of interest, our analysis of *cis*-regulatory elements for the genes linked to the biosynthesis of GSLs and phenolics over-represented the transcriptional recognition sites for MYC and MYB (Figure 7 and Figure 8, Supplementary Table S3), which lays a foundation for functional validation. Future investigation of the transcription factors of these rapeseed cultivars including MYC and MYB will provide a better understanding of the regulation of biosynthetic genes that leads to the changed metabolites.

Plant phenolic compounds are synthesized mainly from the pentose phosphate pathway and the shikimate pathway [53,54]. The patterns of gene expression associated with the metabolic pathway of phenolic compounds were largely positively correlated, especially in the later steps of the biosynthetic pathway [55]. In the current study, we found that the examination of expression for the 13 genes immediately associated with the biosynthetic pathway of phenolic compounds was positively correlated (Figure 2 and Figure 4, and Supplementary Figure S3). Specifically, the highest metabolic levels of rutin and benzoic acid were positively correlated, both transcriptionally and metabolically (Figure 2 and Figure 4, and Supplementary Figure S3). Aside from the early biosynthetic genes that lead to the accumulation of benzoic acid in the seven cultivars, gene expression analysis of our study suggests that 4-coumarate CoA ligase (*4CL*) furcates and leads to the phenylpropanoid pathway responsible for the accumulation of the highest levels of rutin and epicatechin in the seven Korean cultivars (Supplementary Figure S3).

In summary, our results represent the unique signatures of GSLs and phenolic compounds embedded in the seven Korean rapeseed cultivars. In addition, analyses of gene expression and *cis*-regulatory elements of genes related to the biosynthesis of GSLs and phenolic compounds lay fundamental foundations for future studies, and importantly to improve the functionality of the Korean rapeseed cultivars.

## 4. Materials and Methods

### 4.1. Plant Materials and Growth Conditions

The seeds of seven rapeseed cultivars were obtained from Bioenergy Crop Research Institute, Rural Development Administration (Muan-gun, Korea). The sterilized seeds were imbibed in the tap water for 24 h and 200–300 seeds were sowed in the pots with vermiculite soil. The seeds were germinated and grown under a long-day condition (16-h light/8-h dark) photoperiod with a high-intensity level of radiation (flux rate of 50 µmol/s·m^2^) at 25 °C. After 10 days of germination, sprouts of each cultivar were harvested in nitrogen (N) liquid. The frozen tissues were lyophilized and then ground into a fine powder for HPLC analysis and further experimentations.

### 4.2. Extraction and Determination of Glucosinolates

Determination and analysis of GSLs were performed as previously described in [30,56,57] with slight modifications. For extraction, a mini-column equipped with DEAE Sephadex A-25 (GE Healthcare, Uppsala, Sweden) was used. Briefly, 100 mg of ground powder samples was added with 1.5 mL of boiled 70% methanol (*v/v*). For inactivation of *myrosinase*, the mixture was incubated for 5 min in a water bath at 70 °C followed by centrifugation at 12,000× *g* for 15 min at 4 °C, and the supernatant was placed in a new tube. The samples were extracted two more times in the same way. The desulfation reaction was performed overnight at ambient temperature, and then the desulfoglucosinolate was eluted using 0.5 mL of HPLC-grade water. The extracted GSLs were analyzed by using the HPLC system (Agilent Technologies 1200 series, Santa Clara, CA, USA) coupled with an Inertsil ODS-3 reversed-phase column (GL Science, Tokyo, Japan) connected to an E-type cartridge guard column. The column temperature, detected wavelength, and the flow rate were 40 °C, 227 nm, and 0.2 mL/min respectively, with the mobile phase solvent A (HPLC grade ultra-pure water) and solvent B (Acetonitrile). The gradient program was similar to that described by Yeo et al. [56]. Finally, desulfoglucosinolate was quantified relative to the response factor. Extraction of each sample was carried out in triplicates.

### 4.3. Determination of Phenolic Compounds

Approximately 100 mg of ground powder of all samples was extracted separately using 80% (*v/v*) methanol. The samples were sonicated for 60 min while vortexing every 20 min throughout incubation and then centrifuged at 12,000 rpm for about 15 min. The crude extract was transferred to a fresh sterile tube and the remaining sludge was further extracted at least two more times. The extract was filtered and sterilized by using 0.45 μm filters, and then subjected to HPLC analysis. The phenolic compounds were separated by using the HPLC system (Agilent Technologies 1200 series, Santa Clara, CA, USA) coupled with the C_18_ column (RStech Co., Daejeon, Korea). The column temperature, detected wavelength, and the flow rate were 30 °C, 280 nm, and 1.0 mL/min respectively, with an injection volume of 20 µL. The mobile phase consisted of solvent A HPLC grade ultra-pure water/CH_3_COOH (99.85:0.15 *v/v*) and solvent B 100% MeOH. The gradient program was similar to that described by Yeo et al. [56]. For the identification and quantification of phenolic compounds, a spike test was performed by mixing the standard with the extracted sample. Extraction of each sample was carried out in triplicates.

### 4.4. RNA Extraction and Quantitative RT-PCR

Total RNA was extracted using TRIZOL (Invitrogen, Life Technologies, Waltham, MA, USA) reagent. The quality and concentration of total RNA samples were evaluated by agarose gel electrophoresis and a ND-1000 spectrophotometer (NanoDrop, Fisher Thermo, Wilmington, DE, USA). For quantitative RT-PCR (qRT-PCR) analysis, about 1 μg of total RNA was used to synthesize the first-strand complementary DNA using M-MLV reverse transcriptase (Promega, Madison, WI, USA) according to the manufacturer’s instructions. PCR reaction was carried out in a volume of 20 μL that contained 10 μL of BioFACT™ 2X real-time PCR Master Mix (Biofact Co. Ltd., Daejeon, Korea), 4 μL of diluted cDNA, 2 μL of (0.2 μM each) of reverse and forward primers (Supplementary Table S4), and 4 μL of sterilized distilled water. The qRT-PCR was carried out using a Bio-Rad system (Bio-Rad laboratory, Inc., Hungary Ltd., Budapest, Hungary) and the amplification conditions were: 40 cycles consisting of 95 °C for 30 s, 58 °C for 30 s, 72 °C for 30 s, followed by the initial denaturation at 95 °C for 30 s. Information on the selected genes involved in the biosynthesis of GSLs and phenolic compounds are: *BnFMO-OX-2* (XM_013788930), *BnAOP2* (XM_013879976), *BnGSL-OH* (XM_009134650, *BnCYP81F1* (XM_013845052), *BnCYP81F2* (XM_013811596), *BnCYP81F3* (XM_013799561), *BnIGMT1* (XM_013797956), *BnIGMT2* (XM_013797956), *BnPAL* (XM_013811519), *BnBALDH* (XM_013842812), *BnC4H* (DQ485129), *BnC3HS* (XM_022698630), *BnCOMT* (KX944689), *BnC4L* (XM_013882471), *BnCHS* (GQ983005), *BnF3H* (DQ288238), *BnSDH* (XM_013838201), *BnFLS2* (XM_013860217), *BnUFGT* (XM_013794362), *BnLAR* (XM_013791897), *BnANR* (XM_013791897), and *BnACT2* (XM_013881014). Expression of each gene represents the value relative to rapeseed *BnACT2* from the mean *±* standard deviation of three different biological replicates. The primer sequences of all GSLs and phenolics biosynthetic pathway genes analyzed in qRT-PCR are provided in Supplementary Table S4.

### 4.5. Statistical and In Silico Analyses for the GSLs and Phenolics

Mean values of three biological replicates with their respective standard deviation for the identified GSLs and phenolic compounds were analyzed by analysis of variance (ANOVA) and the cultivar difference by Duncan’s multiple range test (DMRT) using Statistical Analysis Software (SAS, system 9.4, 2013; SAS Institute, Inc., Cary, NC, USA) at *p* < 0.05. Raw data associated with accumulated metabolites were further auto-scaled using MetaboAnalyst 5.0 [58]. Normalized mean values were subjected to hierarchical clustering analysis and correspondent heatmap visualization with the Euclidean distance measurement and Ward algorithm. Identification of *cis*-regulatory elements or motifs for biosynthetic genes for GSLs and phenolic compounds was performed using PlantCARE (http://bioinformatics.psb.ugent.be/webtools/plantcare/html/, accessed on 20 August 2021) and Multiple Em for Motif Elicitation (MEME, Suite 5.3.3).

## Data Availability

Not applicable.

## Supplementary Materials

The following are available online at https://www.mdpi.com/article/10.3390/plants10102027/s1, Figure S1: The total content of secondary metabolites obtained in the seven Korean rapeseed cultivars, Figure S2: Metabolic profiles of GSLs identified in the seven Korean rapeseed cultivars, Figure S3: Metabolic profiles of phenolic compounds identified in the seven Korean rapeseed cultivars, Table S1: Determination of glucosinolates content (μmol/g dry wt.) in the seven Korean rapeseed cultivars, Table S2: Determination of phenolic contents (µg/g dry wt.) in the seven Korean rapeseed cultivars, Table S3: Sequence information of primers used in qRT-PCR, Table S4: Transcription factor (TF) binding sites and cis-regulatory elements identified in the biosynthetic genes of GSLs and phenolics identified in this study.
