# Supplementary material for: Targeted Metabolic and In-Silico Analyses Highlight Distinct Glucosinolates and Phenolics Signatures in Korean Rapeseed Cultivars
Source: Plants (Basel). 2021 Sep 27;10(10):2027. doi: 10.3390/plants10102027 (PMC8537057; doi:10.3390/plants10102027)
Supplement: Supplementary file 1 [file plants-10-02027-s001.zip › plants-1376907-supplementary.pdf]

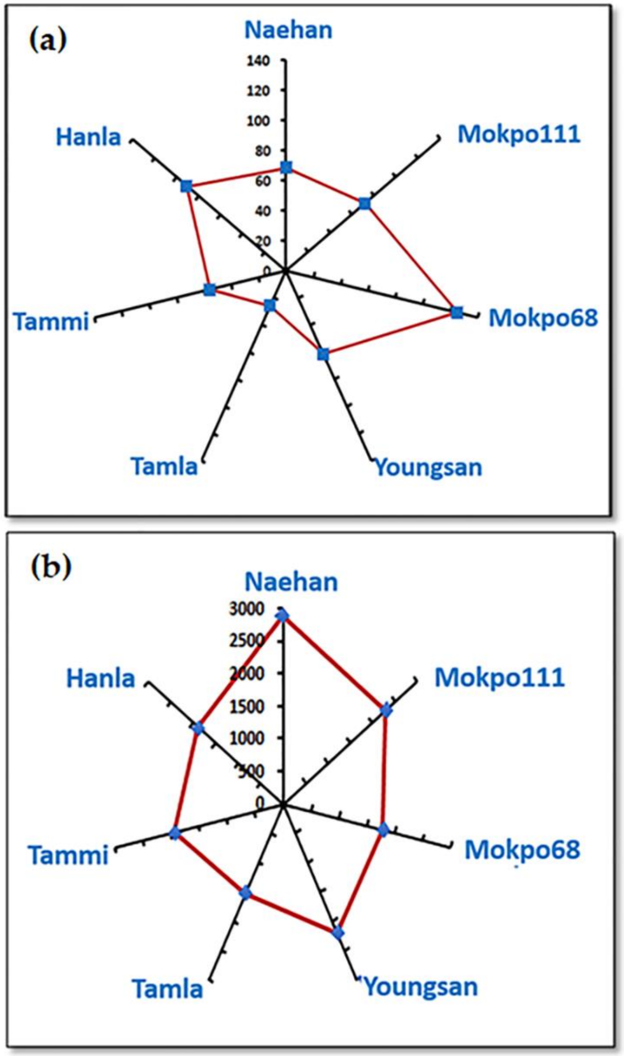

Figure S1. The total level of secondary metabolites obtained in the seven rapeseed cultivars. (**a**) Total GSLs (µmol/g dry wt.) and (**b**) phenolic compounds (µg/g dry wt.) in different cultivars of rapeseed. The obtained results varied significantly ($p < 0.05$), and statistically significant differences were examined according to ANOVA-DMRT.

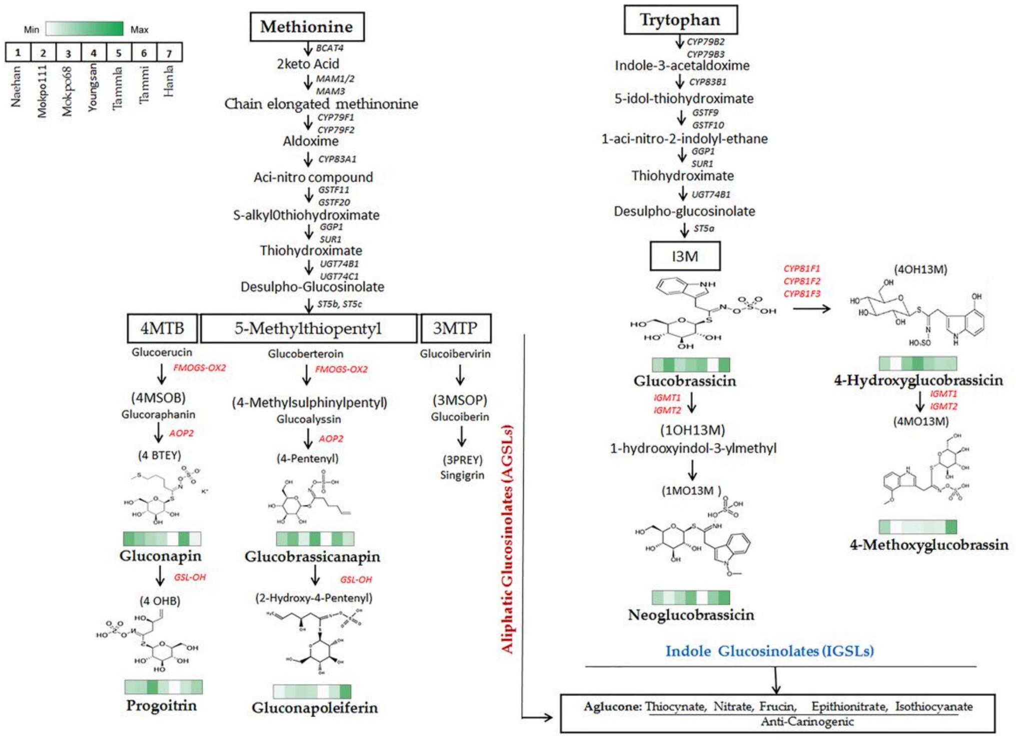

Figure S2. Metabolic profiles of GSLs identified in the seven Korean rapeseed cultivars. Relative levels for each metabolite of the four of AGSLs (progoitrin, gluconapin, gluconapoleiferin, and glucobrassicanapin) and each metabolite of the four of IGSL (4-methoxyglucobrassicin, 4-hydroxyglucobrassicin, neoglucobrassicin, and glucobrassicin) identified in the Naehan (NH), Mokpo111 (M111), Mokpo68 (M68), Youngsan (YS), Tamla (TL), Tammi (TM), and Hanla (HL) are compared between cultivars. Relative abundance of the metabolite between the cultivars for each metabolite was color-scaled. Expressions of selected biosynthetic genes immediately associated with the changed metabolites are color-coded in red.

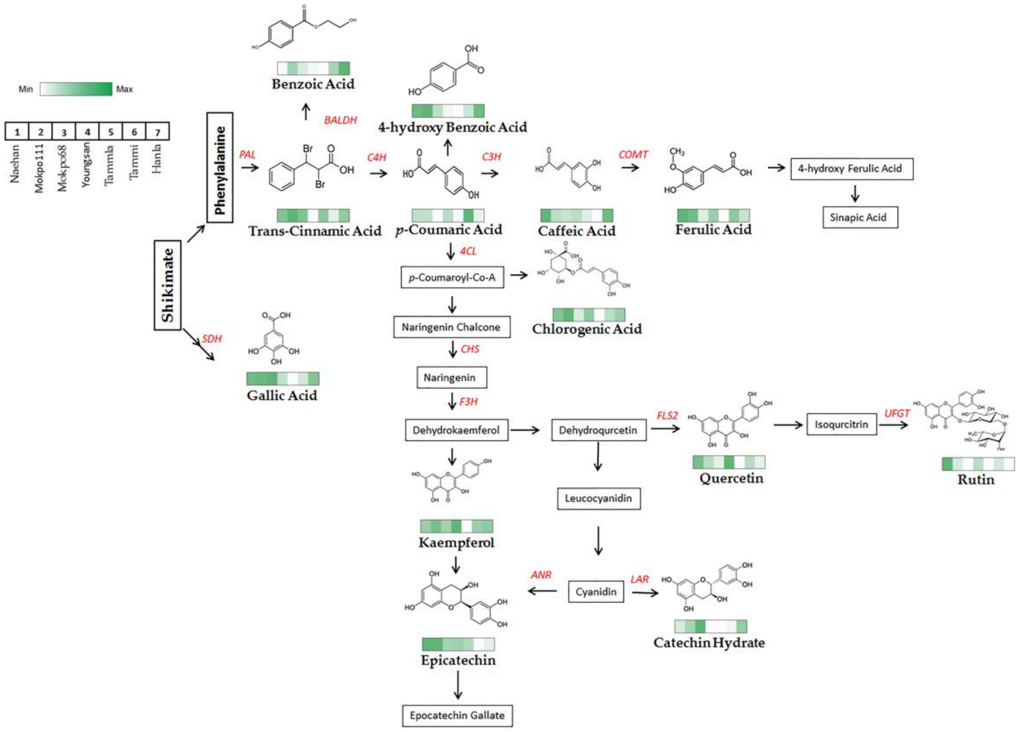

Figure S3. Metabolic profiles of phenolic compounds identified in the seven Korean rapeseed cultivars. Relative levels for each metabolite of the 13 phenolic compounds (chlorogenic acid, catechin hydrate, 4-hydroxybenzoic acid, gallic acid, ferulic acid, *p*-coumaric acid, epicatechin, caffeic acid, rutin, quercetin, *trans*-cinnamic acid, benzoic acid, and kaempferol) identified in the Naehan (NH), Mokpo111 (M111), Mokpo68 (M68), Youngsan (YS), Tamla (TL), Tammi (TM), and Hanla (HL) are compared between cultivars. Relative abundance of the metabolite between the cultivars for each metabolite was color-scaled. Expressions of selected biosynthetic genes immediately associated with the changed metabolites are color-coded in red.

Table S1. Determination of total glucosinolates (μmol/g dry wt.) in different cultivars of rapeseed.

No.	GSL	Cultivars						
		**Naehan**	**Mokpo111**	**Mokpo68**	**Youngsan**	**Tamla**	**Tammi**	**Hanla**
1	**Progoitrin**	48.1 ± 8.91cd	52.03 ± 6.11bc	106.5 ± 11.64a	39.83 ± 2.86d	8.23 ± 1.44e	38.25 ± 2.49d	61.29 ± 5.71b
2	**Gluconapoleiferin**	0.62 ± 0.12c	1.33 ± 0.16b	1.56 ± 0.14b	1.45 ± 0.09b	0.3 ± 0.08c	1.33 ± 0.08b	3.75 ± 0.42a
3	**Gluconapin**	1.83 ± 0.20a	1.38 ± 0.12b	1.05 ± 0.05c	0.89 ± 0.04c	0.33 ± 0.11d	1.91 ± 0.12a	0.49 ± 0.03d
4	**4-Hydroxyglucobrassicin**	0.98 ± 0.17cd	0.16 ± 0.01e	1.35 ± 0.16b	1.99 ± 0.23a	1.17 ± 0.08bc	0.88 ± 0.12d	0.95 ± 0.06cd
5	**Glucobrassicanapin**	0.47 ± 0.11c	0.76 ± 0.08b	0.23 ± 0.05d	0.88 ± 0.03a	0.07 ± 0.01e	0.73 ± 0.04b	0.32 ± 0.03d
6	**Glucobrassicin**	5.93 ± 0.33cd	9.24 ± 1.16a	5.83 ± 0.78d	7.06 ± 0.45bc	7.75 ± 0.62b	3.36 ± 0.35e	9.19 ± 0.39a
7	**4-Methoxyglucobrassicin**	5.47 ± 0.47b	2.43 ± 0.32d	3.23 ± 0.56c	3.33 ± 0.30c	3.59 ± 0.41c	3.48 ± 0.40c	8.33 ± 0.42a
8	**Neoglucobrassicin**	5.32 ± 0.48bcd	4.61 ± 0.47cd	5.23 ± 0.77bcd	6.08 ± 0.56ab	4.31 ± 0.69d	5.64 ± 0.50abc	6.40 ± 0.24a
	**Total**	**68.72 ± 10.33cd**	**71.93 ± 8.34c**	**124.99 ± 13.93a**	**61.50 ± 4.42cd**	**25.76 ± 3.00e**	**55.58 ± 3.20d**	**90.71 ± 6.85b**

- Different letters in the same rows varied significantly ($p < 0.05$). Results are the average of three different biological replications.

Table S2. Determination of total phenolic content (μg/g dry wt.) in different cultivars of rapeseed.

No.	Phenolics	Cultivars						
		**Naehan**	**Mokpo111**	**Mokpo68**	**Youngsan**	**Tamla**	**Tammi**	**Hanla**
1	**Gallic Acid**	80.10 ± 2.95ab	82.27 ± 4.2ab	84.91 ± 1.06a	58.38 ± 3.26c	43.18 ± 2.16d	54.31 ± 3.35c	74.72 ± 8.23b
2	**4-hydroxybenzoic Acid**	81.22 ± 5.33a	86.61 ± 7.8a	48.75 ± 3.07b	29.77 ± 3.32c	25.44 ± 8.79c	43.86 ± 1.55b	81.09 ± 2.63a
3	**Catechin Hydrate**	59.01 ± 1.94c	77.35 ± 10.28b	107.25 ± 5.63a	44.7 ± 1.58d	44.62 ± 5.49d	47.97 ± 3.94d	84.51 ± 2.06b
4	**Chlorogenic Acid**	69.51 ± 2.74ab	75.95 ± 4.55a	54.55 ± 3.44de	67.26 ± 5.23bc	48.77 ± 4.01e	59.11 ± 1.1cd	64.55 ± 7.85bc
5	**Caffeic Acid**	41.37 ± 3.16a	26.2 ± 4.35b	24.23 ± 0.94bc	26.08 ± 1.81b	20.77 ± 3.47c	16.02 ± 0.41d	39.27 ± 0.87a
6	**Epicatechin**	484.97 ± 41.48a	483.42 ± 61.16a	352.93 ± 15.02b	363.92 ± 14.95b	333.67 ± 40.38b	197.89 ± 9.98c	241.14 ± 42.83c
7	***p*-coumaric Acid**	66.33 ± 1.69c	65.1 ± 2.28c	38.13 ± 0.06e	69.78 ± 0.33b	41.27 ± 3.94e	112.83 ± 1.74a	47.17 ± 0.24d
8	**Ferulic Acid**	33.59 ± 1.78a	32.27 ± 3.6ab	24.17 ± 1.97d	28.92 ± 0.47bc	21.99 ± 2.53d	29.86 ± 1.32ab	25.79 ± 2.34cd
9	**Benzoic Acid**	317.33 ± 11.65c	426.86 ± 36.65b	354.15 ± 46.5c	322.47 ± 41.56c	308.92 ± 38.72c	421.28 ± 45b	522.67 ± 6.83a
10	**Rutin**	1292.46 ± 32.99a	616.99 ± 60.07c	432.32 ± 3.28d	781.83 ± 16.65b	419.05 ± 14.8d	644.83 ± 7.45c	432.77 ± 9.73d
11	**Trans-cinnamic acid**	15.35 ± 0.36ab	16.91 ± 4.08a	15.5 ± 3.38ab	10.69 ± 1.69c	15.15 ± 1.36ab	11.59 ± 0.46bc	14.85 ± 0.76ab
12	**Quercetin**	206.41 ± 1.06a	146.69 ± 26b	111.83 ± 35.13c	226.8 ± 19.92a	90.49 ± 12.64c	147.58 ± 10.06b	108.09 ± 3.46c
13	**Kaempferol**	146.19 ± 16.01ab	161.01 ± 21.57a	143.59 ± 31.43ab	171.45 ± 16.44a	108.09 ± 9.45b	146.05 ± 24.11ab	150.46 ± 20.12a
	**Total**	**2893.84 ± 77.01a**	**2297.63 ± 102.88b**	**1792.31 ± 68.56d**	**2202.05 ± 4.43b**	**1521.42 ± 30.21e**	**1933.17 ± 27.31c**	**1887.08 ± 76.45cd**

- Different letters in the same rows varied significantly ($p < 0.05$). Results are the average of three different biological replications.

Table S3. Transcription factor (TF) binding sites and *cis*-regulatory elements identified in the biosynthetic genes of GSLs and phenolics identified in this study.

No.	Category	Metabolites	
		**Elements in GSLs**	**Elements in phenolics**
1	**General Transcription**	TATA-box	TATA-box
		CAAT-box	CAAT-box
2	**Hormone**	P-box (gibberellin-responsive)	P-box (gibberellin-responsive)
		GARE-motif (gibberellin-responsive)	
		TGA-element (auxin-responsive)	TGA-element (auxin-responsive)
		ABRE (abscisic acid-responsive)	ABRE (abscisic acid-responsive)
		GTCA-motif (JA-responsive)	GTCA-motif (JA-responsive)
		TGACG-motif (JA-responsive)	
3	**Stress**	ARE (anaerobic-responsive)	ARE (anaerobic-responsive)
		AE-box (light-responsive)	AE-box (light-responsive)
		ATC-motif (light-responsive)	ATC-motif (light-responsive)
			ATCT-motif (light-responsive)
		G-box (light-responsive)	G-box (light-responsive)
			GA-motif (light-responsive)
		T1-motif (light-responsive)	
			I-box (light-responsive)
			MRE (MYB binding site and light-responsive)
		TCCC-motif (light-responsive)	
		TCT-motif (light-responsive)	TCT-motif (light-responsive)
		LTR (low temperature-responsive)	
4	**TF**	MYC	MYC
		MYB	
		Myb binding site	MBS (MYB binding site)
5	**ETC**		AT-rich DNA-binding protein-related
			O2-site (zein metabolism regulation-related)

Table S4. List of gene-specific primers used for qRT-PCR.

Primer Name	Primer sequence 5′–3′	Product length (bp)
	**GSLs biosynthetic genes**	
BnFMO-OX-2-F	GCCATGCTTGAAGCGTTAGGTAT	139
BnFMO-OX-2-R	AAGGCTCAACAGGCTGACAA	
BnAOP2-F	TTGACTATGCGTACGTTTCTCT	110
BnAOP2-R	GGAGTGGTGCCACATCAATA	
BnGSL-OH-F	CCTCCCAAGTACAGAGACACC	131
BnGSL-OH-R	GCTTAGCGAAGCATTCCCAAC	
BnCYP81F1-F	GGCGCTAGGATCGTTGATT	101
BnCYP81F1-R	GATCCATTTTGCGCATACCC	
BnCYP81F2-F	AGAGTATGATGCTTGCGGG	100
BnCYP81F2-R	GCTTTCGCCTTCTTCAACAC	
BnCYP81F3-F	TTGGCGTTAGGATCGTTGATT	102
BnCYP81F3-R	GCTCTTTCTTACGCATTGCC	
BnIGMT1-F	CAATGTTCCGGTGGGAAAGA	100
BnIGMT1-R	AGTGATAAGCCTGGCAAACG	
BnIGMT2-F	GTTTGAAGCGTTAGCCGCAG	146
BnIGMT2-R	ACAGCAAGAAGACGAATTGAGAC	
BnACTIN-F	GTATTATGAATGGGATCAAAGTTTC	122
BnACTIN-R	TCACACACAAGTGTATAGAAACGATA	
	**Phenolics biosynthetic genes**	
BnC4H-F	CCACCATCGTAATGAAGCCA	139
BnC4H-R	ATTGCGATATTGGAACGAATCC	
BnPAL-F	AGCCTTCCTCTGTTAATCTGT	112
BnPAL-R	GACACCATTCACATTACACTCA	
BnC3HS-F	GGGGTGTCTTGTTTGGTTTC	108
BnC3HS-R	TGCTGGGAAGTATGTATAGGTT	
BnC4L-F	GAGGGTACGAAAAGTGGCTT	148
BnC4L-R	AATGCATGGAGATTGGCTGA	

BnCHS-F	TTGGACGAGATGAGGAGGAA	118
BnCHS-R	AAGACCACTGTCTCTACGGT	
BnF3H-F	GCGAAGCCTCTAGACCAAAT	114
BnF3H-R	AACTTAAATTCACCCAACATGACA	
BnBALDH-F	ACTTGCAGGTCAAGGCAGTC	132
BnBALDH-R	CCAAGTAACAAACCATGGCCTTA	
BnSDH-F	TGTGGAGCCGTAACAGTGTC	128
BnSDH-R	CACACACAAGCGAGAGAGGT	
BnUFGT-F	ATTATCAATGGAGTCTTCACGAGAG	144
BnUFGT-R	TAATTTGAAGTGCTCACAACTGCT	
BnFLS2-F	GCTCAATAAGCTTCCTCTGG	119
BnFLS2-R	TCCTATCGCGAGAACACAAC	
BnLAR-F	GGATAGCGTCAGTTTCGCAC	109
BnLAR-R	AACTTCCCACAGAAATTGCAAAG	
BnCOMT-F	ACCACCGAAAACTCACATTACA	113
BnCOMT-R	ATGTTCGTCGCTCCAATCGT	
BnANR-F	TCCGAGGATAGCGTCAGTTTC	119
BnANR-R	TCTCCCCACAGAAATTGCAAAA	
